# Neddylation of HER2 Inhibits its Protein Degradation and promotes Breast Cancer Progression

**DOI:** 10.7150/ijbs.75852

**Published:** 2023-01-01

**Authors:** Xiaohong Xia, Tumei Hu, Xiaoyue He, Yuan Liu, Cuifu Yu, Weiyao Kong, Yuning Liao, Daolin Tang, Jinbao Liu, Hongbiao Huang

**Affiliations:** 1 Affiliated Cancer Hospital & Institute of Guangzhou Medical University, Guangzhou 510095, China.; 2 Guangzhou Municipal and Guangdong Provincial Key Laboratory of Protein Modification and Degradation, School of Basic Medical Sciences, Guangzhou Medical University, Guangzhou 511436, China.; 3 Department of Surgery, UT Southwestern Medical Center, Dallas, Texas 75390, USA.

**Keywords:** HER2, neddylation, ubiquitin, degradation, breast cancer

## Abstract

HER2 is a transmembrane receptor with intrinsic tyrosine kinase activity that is overexpressed in almost 25% of human breast cancers. Here, we report that the neddylation of HER2 is a new post-translational modification that controls its expression and oncogenic activity in human breast cancer. Two critical members in the neddylation pathway, NEDD8 and NEDD8-activating enzyme E1 subunit 1 (NAE1), are detected in human breast specimens. Overexpressed NEDD8 and NAE1 are positively correlated with HER2 expression in human breast cancer. Subsequent structure and function experiments show that HER2 directly interacts with NEDD8 and NAE1, whereas HER2 protein expression is decreased by neddylation depletion. Mechanistically, neddylation inhibition promotes the degradation of HER2 protein by improving its ubiquitination. HER2 overexpression abrogates neddylation depletion-triggered cell growth suppression. The inhibition of neddylation synergized with trastuzumab significantly suppresses growth of HER2 positive breast cancer. Collectively, this study demonstrates a previously undiscovered role of NEDD8-dependent HER2 neddylation promotes tumor growth in breast cancer.

## Introduction

Breast cancer ranks first in the incidence and mortality of female malignant tumors in the world 1. It is a heterogeneous disease and includes five subtypes: basal-like, luminal A, luminal B, unclassified and human epidermal growth factor receptor 2 (HER2) 2-4. Among them, the HER2-positive (HER2^+^) phenotype accounts for 25% of patients with breast cancer 5. The protein overexpression and gene amplification of HER2 decrease overall survival associated with poor prognosis 6. The pathological parameters of HER2^+^ breast cancer are usually unfavorable, such as high grade, lymph-node involvement, high cell proliferation rate, and poor differentiation 7. Due to the progress in the application of HER2 targeted therapy, the overall survival of patients with HER2^+^ breast cancer has been prolonged in the past decade 8,9. Although the recurrence and mortality are remarkably decreased 10,11, the resistance to HER2 targeted drugs (such as trastuzumab) remains a major clinical challenge for the treatment of breast cancer patients 12. After receiving trastuzumab and adjuvant chemotherapy, approximately 15% of patients relapse, and 70% of patients with HER2^+^ breast cancer will inevitably acquire therapy resistance 13. In addition to understanding the mechanisms of acquired resistance, there is an urgent need to develop new molecular targets to treat patients with HER2^+^ breast cancer.

HER2, encoded by the oncogene Erbb2 and acts as an orphan receptor, belongs to the family of epidermal growth factor receptor (EGFR). The EGFR family is often considered the “prototypical” receptor tyrosine kinase (RTK), which includes four members: HER1 (EGFR), HER2, HER3 and HER4^14^. After binding to growth factors, HER2 is phosphorylated, leading to activation of downstream kinases for intracellular signal transduction, involving in cell proliferation, survival, migration and polarity changes 15. Therefore, regulating the phosphorylation of HER2 has become a treatment strategy for patients with HER2 positive breast cancer. Alternatively, degrading pathogenic proteins through the ubiquitin proteasome system (UPS) may be a promising therapeutic strategy 16,17. The UPS is a post-translational modification (PTM) that plays a central role in stabilizing protein expression 18. In addition to ubiquitination, PTM of proteins also has many forms, such as phosphorylation, sumoylation, neddylation, acetylation, glycosylation and methylation. These different PTMs can cross-talk with ubiquitination to shape protein expression and function. Although the stabilize of HER2 regulated by the UPS has been demonstrated 19,20, how other PTMs affect HER2 ubiquitination is still poorly understood.

Neddylation, a process of removing the ubiquitin-like protein (NEDD8) onto the target substrate, is a new type of PTM, similar to ubiquitination 21. The neddylation consists of NEDD8-activating enzyme E1 subunit 1 (NAE1; an activating enzyme), conjugating enzymes, and various E3 ligases 22. The system primarily focus on the activation of Cullin-RING ubiquitin ligases, but the NEDD8 has identified an increasing number of targets. Neddylation is reversible by NEDP1 and JAB1/CSN5, which act as deneddylases 23,24. The inhibitor of NAE1, pevonedistat/MLN4924, has been used in phase II clinical trials to improve response to chemotherapy or radiotherapy 25,26. Functionally, neddylation is an important regulator of cell viability and tissue development. The aberrant neddylation upregulation is closely related to the pathogenesis of diseases, such as Alzheimer disease and cancer 27,28, and has become an emerging therapeutic target.

Here, we demonstrate that HER2 is a key neddylation substrate in breast cancer cells. HER2 neddylation reduces UPS-mediated degradation to stabilize HER2 protein expression. Consequently, HER2 neddylation promotes the progression of HER2^+^ breast cancer cells *in vitro*. These findings indicate that neddylation may be a new therapeutic target for HER2^+^ breast cancer.

## Results

### Neddylation positively correlates with the development of HER2 positive breast cancer

**Figure 1:**
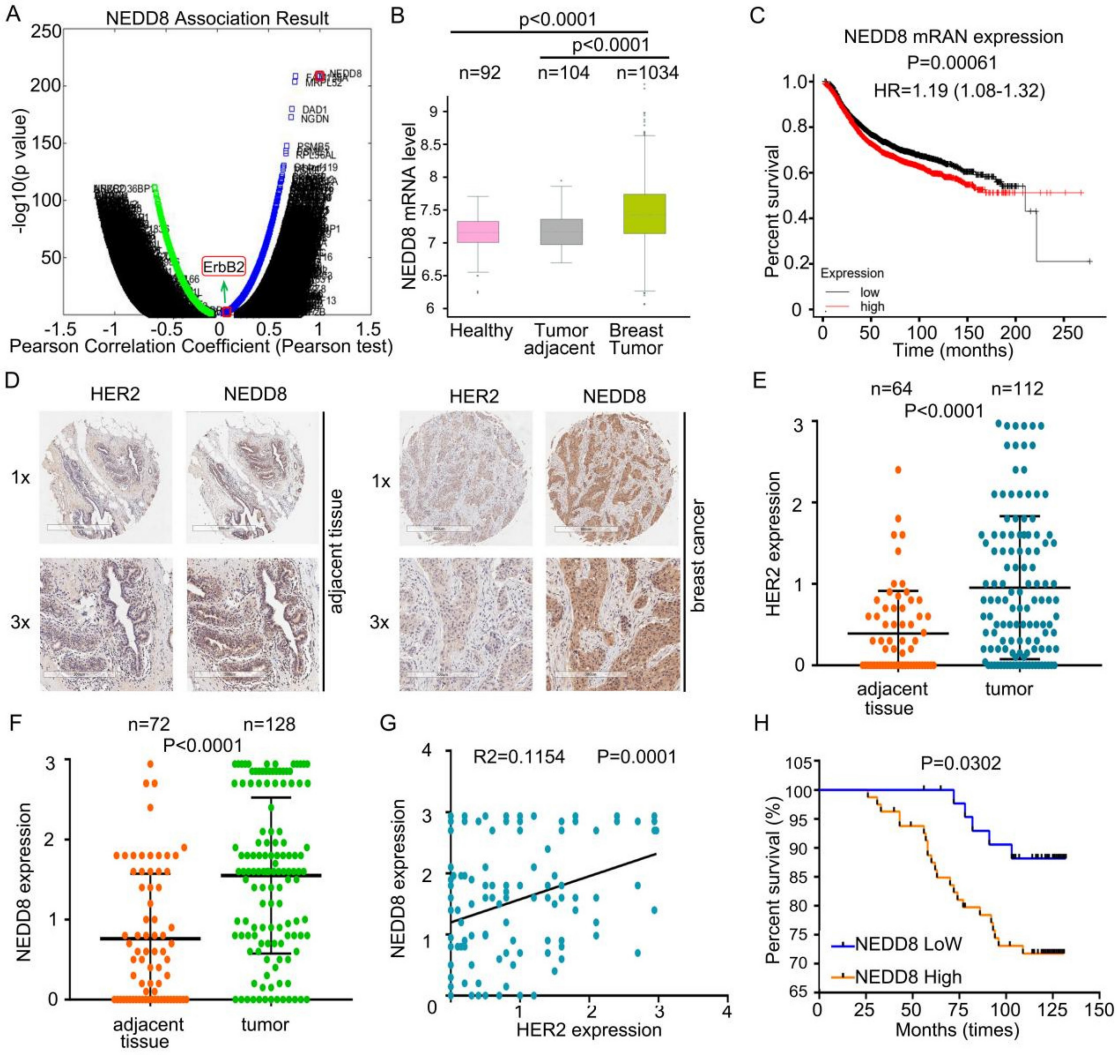
** Neddylation positively correlates with the development of HER2 positive breast cancer. A** Identified genes associated with NEDD8 in the LinkedOmics web application (http://www.linkedomics.org). **B** The mRNA level of NEDD8 in human clinical specimens searching from the published microarray dataset. **C** According to NEDD8 level, Kaplan-Meier curves of breast cancer patients. **D** Immunohistochemical staining using anti-HER2 and anti-NEDD8 antibodies in human clinical breast cancer tissue and adjacent tissues arrays.** E** The HER2 and (**F**) NEDD8 expressions were read and analyzed. **G** The correlation between HER2 and NEDD8 expression. **H** According to NEDD8 protein expression, Kaplan-Meier curves of breast cancer patients.

To allow the gene identification modified with NEDD8, we first search genes that interact with NEDD8 using multi-omics analysis3 29. Surprisingly, ErbB2, an oncogene, was identified as one of these candidate genes (Pearson correlation=0.0916; P value=0.0024) (Figure 1A)**.** We speculated neddylation cloud play a critical role in HER2 positive breast cancer. To explore the clinical significance of neddylation activation, we first used the published microarray dataset 30 and compared the expression patterns of NEDD8 in healthy, breast cancer tumor, and adjacent tissues. In breast cancer tissues, NEDD8 mRNA levels were higher than healthy tissues and adjacent tissues (Figure 1B). In addition, patients with NEDD8 overexpression had a lower overall survival in all breast cancer (Figure 1C) 31. Therefore, analysis of these published gene expression data indicates a potential role of NEDD8 in controlling HER2^+^ breast cancer.

Next, we recruited breast cancer patients to detect the expression of NEDD8 and HER2 proteins in human breast cancer (Figure 1D). Consistent with mRNA analysis, the results of immunohistochemistry revealed that HER2 and NEDD8 were highly expressed in breast cancer tissues (Figures 1E and F). Importantly, we found that NEDD8 and HER2 protein expression were positively correlated (Figure 1G). After analyzing the patient's survival rate, NEDD8 was identified as an independent poor prognostic factor in all breast cancer (Figure 1H). Collectively, these findings suggest that up-regulated neddylation is closely related to the development of HER2^+^ breast cancer patients.

### Neddylation regulates HER2 protein degradation

**Figure 2:**
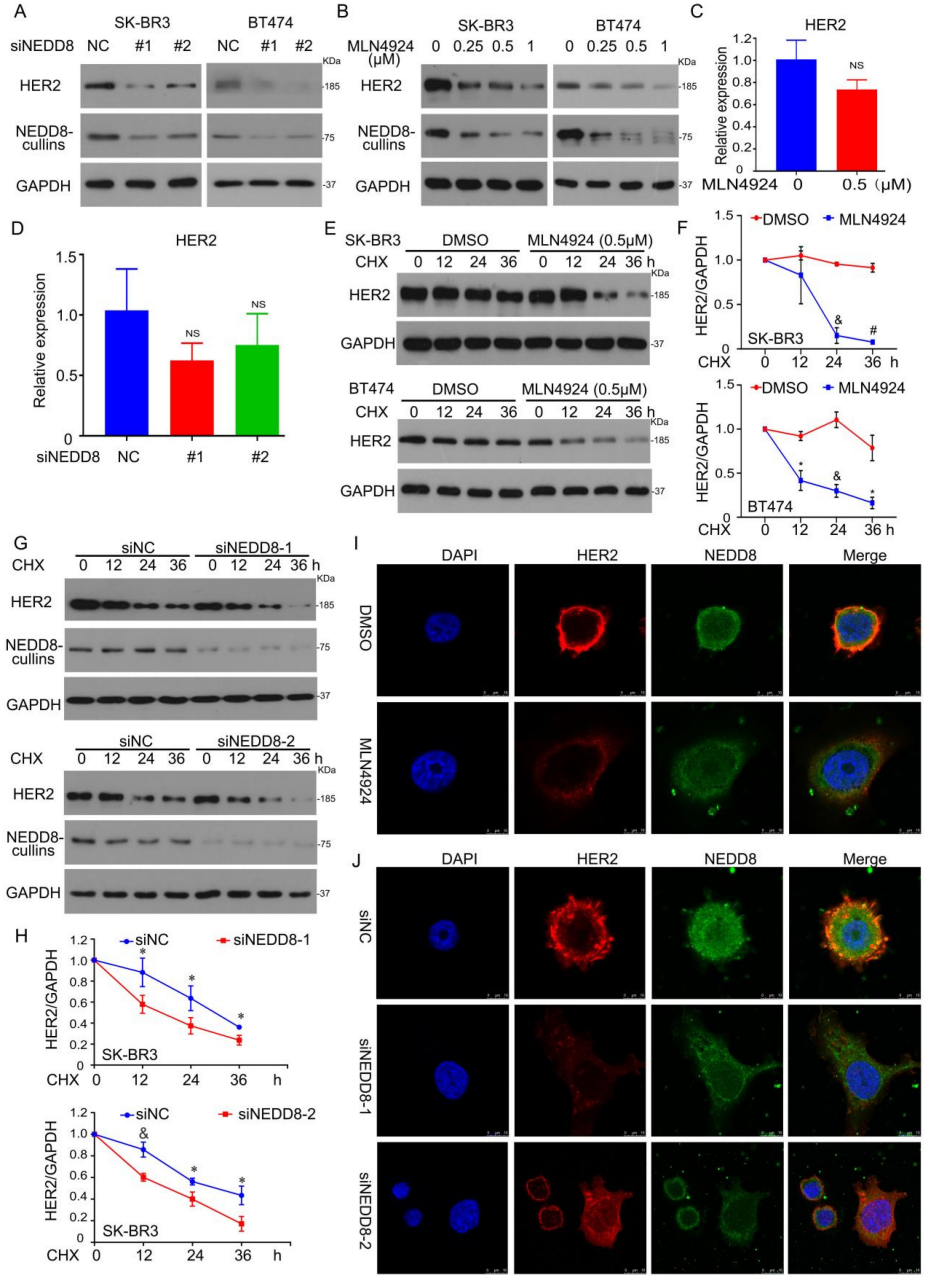
** Neddylation regulates HER2 protein degradation. A** NEDD8 siRNA was used to treat cells for 96 h, subjected to western blot assay for HER2 and NEDD8-cullins expression. **B** MLN4924 treated cells for 24 h, followed by western blot assay for HER2 and NEDD8-cullins expression. Cells were treated to (**C**) MLN4924 for 24 h and (**D**) NEDD8 siRNA for 96 h, respectively. And then total RNA was collected for HER2 level by RT-qPCR. ns represents no significant. **E** SK-BR3 and BT474 cells were treated with MLN4924 and CHX (50 μg/ml) for HER2 protein expression. **G** SK-BR3 and BT474 cells were treated with NEDD8 siRNA and CHX (50 μg/ml) for HER2 protein expression. **F**, **H** The bands of HER2 quantified by densitometry with Image J. ^*^P<0.05, ^&^P<0.01, ^#^P<0.001. SK-BR3 cells were treated to (**I**) MLN4924 and (**J**) NEDD8 siRNA, respectively, subjected to confocal microscope for HER2 and NEDD8 locations and expressions.

NEDD8 is a ubiquitin-like molecule that can covalently bind to certain cellular proteins. To determine whether HER2 is a substrate protein of NEDD8, we first used RNAi to inhibit NEDD8 expression in two HER2-positive breast cancer cell lines (BT474 and SK-BR3). Indeed, silencing NEDD8 leaded to down-regulation of HER2 protein expression (Figure 2A). We also used the E1 inhibitor MLN4924 to treat cells and observed HER2 expression. Consistently, MLN4924 inhibited the expression of HER2 protein (Figure 2B), further confirmed that the stability of HER2 protein can be regulated at multiple levels.

Protein levels are affected by transcription synthesis and protein degradation. We next analyzed whether NEDD8 also affects HER2 gene transcription. RT-qRCR analysis of HER2 mRNA showed that MLN4924 had no effect on transcription synthesis of HER2 (Figure 2C). In wild-type and NEDD8-knockdown cells, HER2 gene expression have no different (Figure 2D). Therefore, we hypothesized that NEDD8-mediated neddylation may directly mediate the degradation of HER2 protein.

To test this hypothesis, we measured HER2 protein half-life in in BT474 and SK-BR3 cells by cycloheximide (CHX) chase assay. Like the administration of MLN4924 (Figures 2E and F), the knockdown of NEDD8 also induced a reduction in the protein half-life of HER2 (Figures 2G and H). In supplementary Figure 2, The treatment of CHX up to 24 hours under EGF treatment also triggered HER2 degradation. These findings indicate that neddylation inhibition promotes the degradation of HER2 protein. Given that HER2 is a membrane protein, we next explored whether neddylation translocates with HER2. Image analysis found that MLN4924 decreased the expression of HER2 in the membrane and did not translocate with it (Figure 2I). Similar results were observed in NEDD8-knockdown SK-BR3 cells (Figure 2J).

### NEDD8-cullins interacts with HER2

**Figure 3:**
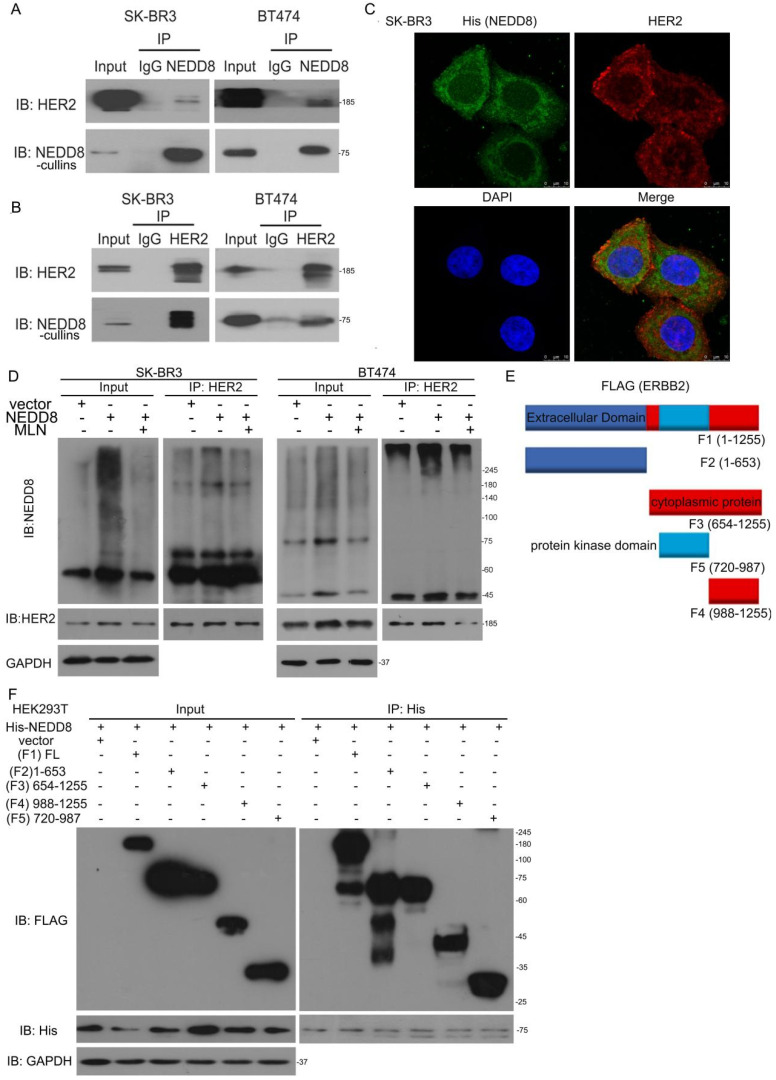
** NEDD8-cullins interacts with HER2. A** Cell lysis was extracted from SK-BR3 and BT474 cells. Immunoblot analysis of anti-NEDD8 immunoprecipitate. **B** Immunoblot analysis of anti-HER2 immunoprecipitate. **C** SK-BR3 cells were transfected with His-tagged NEDD8. Confocal microscope was applied to test HER2 and NEDD8 locations. **D** Cells were transfected with His-tagged NEDD8 and MLN4924 treatment. Immunoblot analysis of anti-HER2 immunoprecipitate for NEDD8 expression. **E** A schematic diagram of HER2 is shown.** F** Cells were transfected with His-tagged full-length NEDD8 and FLAG-tagged full length and truncated plasmids of HER2. Immunoblot analysis of anti-His immunoprecipitate for FLAG expression.

To define the potential molecular mechanism of neddylation-related degradation of HER2 protein, co-immunoprecipitation (Co-IP) combined with Western blot analysis was used to identify NEDD8-cullins interacting proteins. Co-IP using antibodies against NEDD8-cullins or HER2 revealed the strong interaction of NEDD8-cullins with HER2 in BT474 and SK-BR3 cells (Figure 3A and B). In addition to biochemical analysis, image analysis showed that His-tagged NEDD8 was co-localized with HER2 in SK-BR3 cells (Figure 3C). To further detect this modification, Co-IP under NEDD8 ectopically expressed was applied. HER2 migrated to high molecular-weight expression and MLN4924 impaired the neddylation of HER2 (Figure 3D). Cell-free assays *in vitro* demonstrate a protein as substrated for enzyme-mediated post-translational modification using purified proteins. In this scenario, we judged whether HER2 can happen this phenomenon. Incubation of purified protein expressed HER2 with E1, E2 and NEDD8 with ATP induced the appearance of slower-migrating band than without ATP (Supplementary Figure 3A). Overall, these findings indicate that HER2 can be neddylated by NEDD8.

To clarify the structural basis of NEDD8-mediated HER2 neddylation, we generated several HER2 truncated mutants, including extracellular domain (1-654 aa), cytoplasmic domain (676-1255 aa), protein domain (720-987 aa), domian (988-1255 aa) (Figure 3E). Surprisingly, all four domains of HER2 interacted with NEDD8 (Figure 3F). We speculated that neddylation may not only affect HER2 ubiquitination, but also its phosphorylation or/and other functions.

### Neddylation inhibits HER2 polyubiquitination via K48 linkage

**Figure 4:**
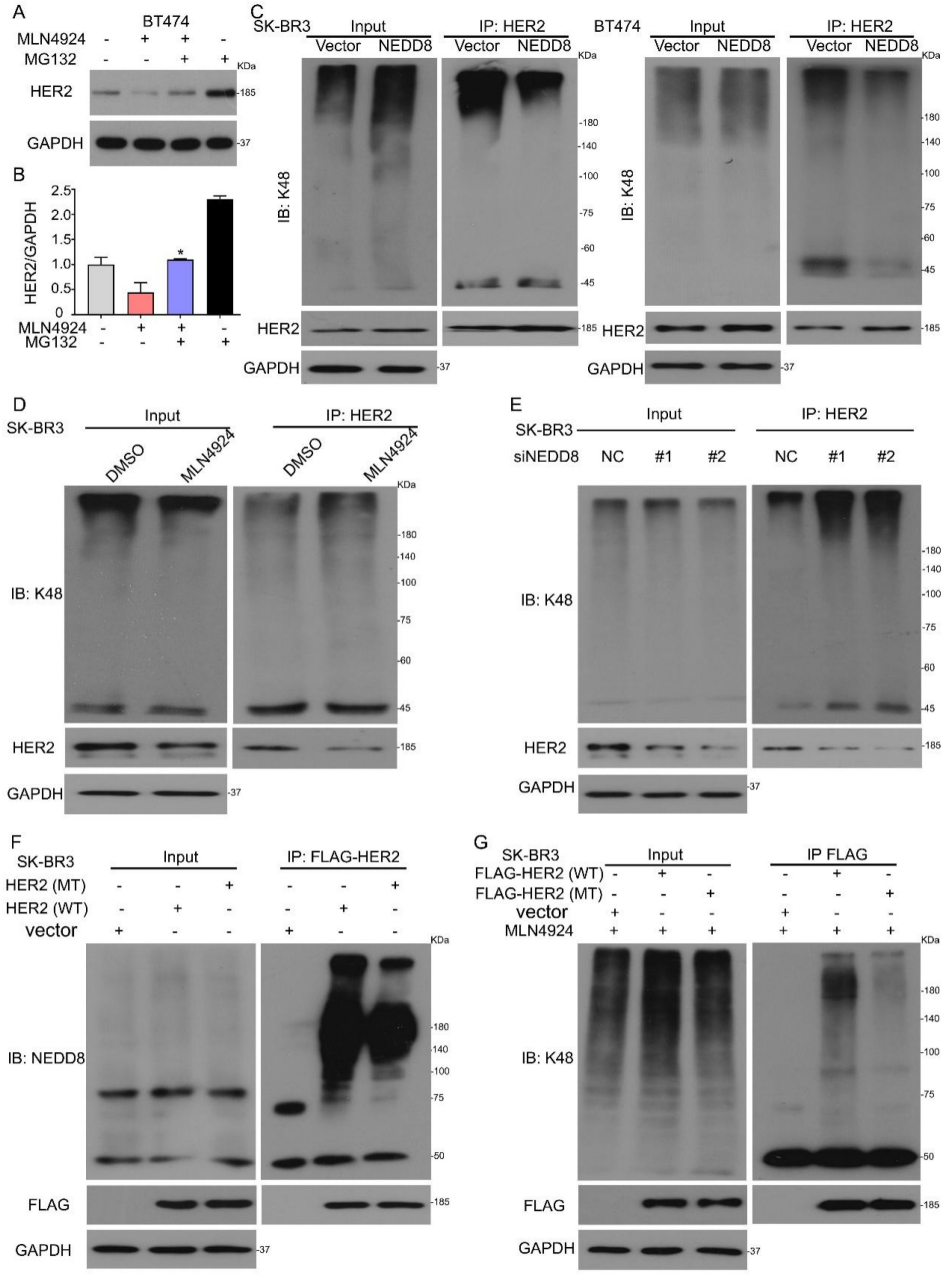
** Neddylation inhibits HER2 polyubiquitination via K48 linkage. A** MLN4924 treated BT474 cells for 18 h and then added MG132 for the additional 6 h, subjected to western blot for HER2 expression. **B** The bands of HER2 were calculated. ^*^P<0.05. SK-BR3 and BT474 cells were treated with (**C**) His-tagged NEDD8, (**D**) MLN4924 and (**E**) NEDD8 siRNA, respectively. Immunoblot analysis of anti-HER2 immunoprecipitate for K48 expression. **F** SK-BR3 cells were treated with FLAG-tagged HER2 and its deletion mutant, Immunoblot analysis of anti-HER2 immunoprecipitate for NEDD8 expression. **G** SK-BR3 cells were treated with FLAG-tagged HER2 and its deletion mutant, and then treated with MLN4924 for 24 h. Immunoblot analysis of anti-HER2 immunoprecipitate for K48 expression.

Several previous studies have shown that HER2 is degraded in a ubiquitin-dependent manner 19,20. However, these studies do not rule out the possible role of K48 polyubiquitination in HER2 degradation. To determine whether HER2 is subjected to polyubiquitylation by neddylation, we used MG132, a classical proteasome inhibitor. Western blot analysis showed that MG132 reversed MLN4924-mediated HER2 protein degradation (Figures 4A and B). To investigate whether neddylation mediates the ubiquitination of HER2, we applied Co-IP and Western blot analysis in BT474 and SK-BR3 cells. When cells were transfected with His-NEDD8, K48 polyubiquitinated HER2 was reduced (Figure 4C). To further prove that HER2 is a substrate of neddylation, we compared the polyubiquitination of HER2 by DMSO or MLN4924, respectively. While the neddylation inhibition promoted the HER2 polyubiquitylation (Figure 4D and Supplementary Figure 3B, C). HER2 was polyubiquitinated by NEDD8 siRNA (Figure 4E and Supplementary Figure 3D, E). These findings confirm a significant role of NEDD8 in the inhibition of HER2 polyubiquitination.

It was previously shown that the ubiquitination modification site of HER2 is Y1112 19. To determine whether Y1112 is involved in HER2 polyubiquitination under conditions of NEDD8 inhibition, we generated FLAG-tagged full-length HER2 (HER2/WT) and FLAG-tagged inactive mutant of HER2 (HER2/Y1112F, which tyrosine 1112 was mutated to a phenylalanine). The FLAG pulled down assays showed that HER2 mutant reduced the neddylation chain (Figure 4F). We further confirmed this finding by testing HER2 ubiquitination. When the ubiquitination modification site of HER2 was mutated, HER2 K48 polyubiquitination was reduced (Figure 4G). In summary, our data clearly confirmed that neddylation inhibition increases polyubiquitylation of HER2 for subsequent proteasome-mediated degradation via conventional K48 linkage.

### Neddylation inhibition induces growth suppression of HER2^+^ breast cancer cells

**Figure 5:**
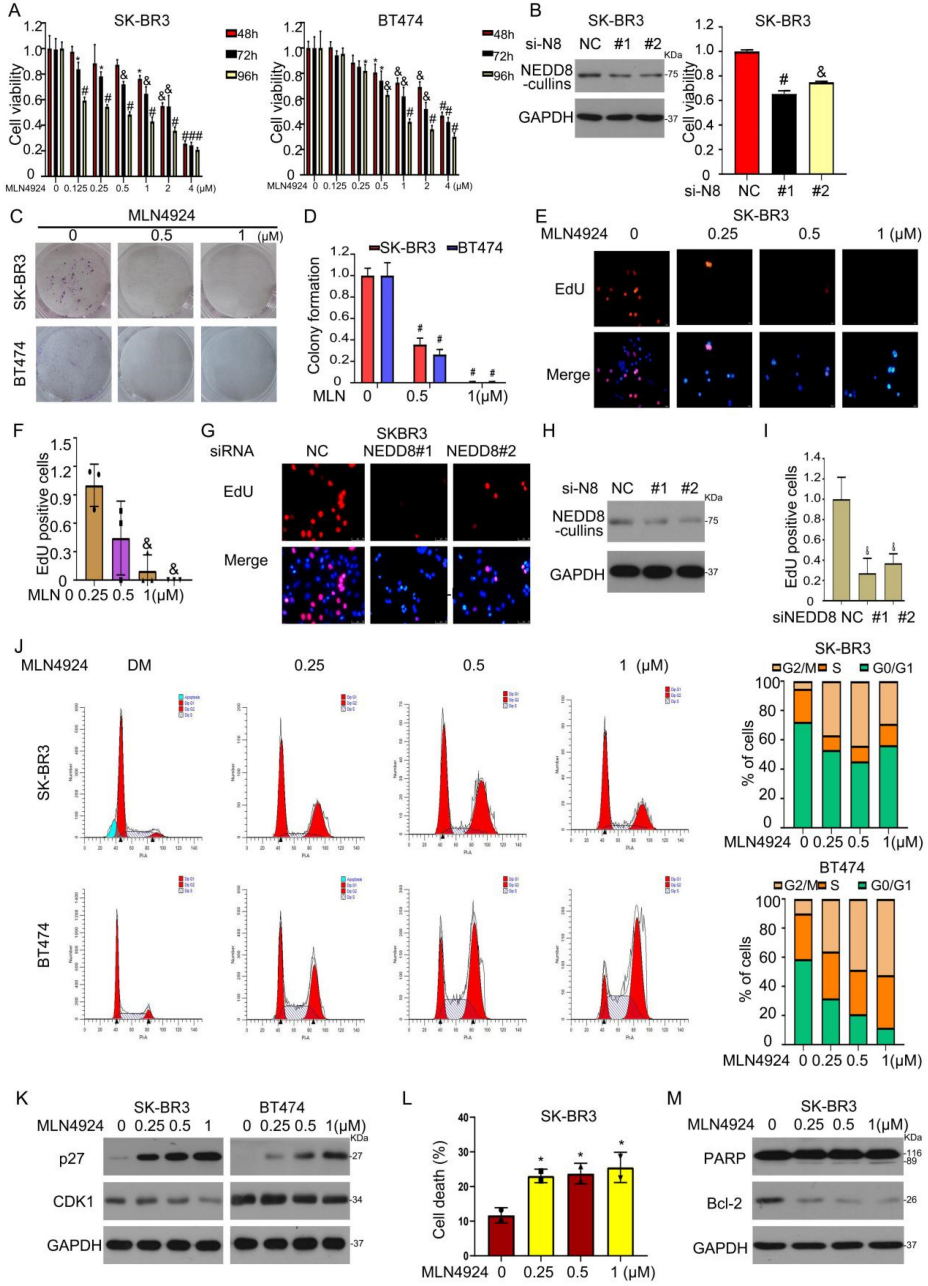
** Neddylation inhibition induces growth suppression of HER2^+^ breast cancer cells. A** MLN4924 treated cells for MTS analysis. **B** NEDD8 siRNA was used to treat cells for western blot and MTS analysis. Cells treated MLN4924 for (**C**) colony formation and (**E**) EdU staining. The number of (**D**) clone cells and (**F**) EdU stained cells was calculated. **G** The cells exposed to NEDD8 siRNA were stained with EdU. **H** Western blot assay for NEDD8 expression. **I** The EdU stained cells was calculated. Cells treated with MLN4924, subjected to flow cytometry and western blot assays for (**J**, **K**) cell cycle and (**L**, **M**) apoptosis. ^*^P<0.05, ^&^P<0.01, ^#^P<0.001.

We next evaluated the biological significance of neddylation in HER2-positive breast cancer. First, the NAE1 inhibitor MLN4924 suppressed cell viability by MTS assay (Figure 5A). Second, siRNA-mediated NEDD8 depletion caused growth inhibition in SK-BR3 cells (Figure 5B). Third, MLN4924 treatment significantly inhibited the clone formation and cell proliferation of two HER2-positive cell lines (Figures 5C-I). Forth, MLN4924 caused cell cycle arrest in G2/M phase in BT474 and SK-BR3 cells (Figure 5J). Western blot assay further confirmed that MLN4924 induced the accumulation of p27 and downregulated the expression of CDK1 (Figure 5K), which are cell cycle regulators. Finally, flow cytometry assay validated that MLN4924 led to apoptosis (Figures 5L and M). These experiments demonstrated that the inhibition of neddylation by MLN4924 can lead to cell cycle arrest and apoptosis.

We also investigated whether MLN4924 enhances the anti-cancer activity of trastuzumab (Herceptin), which is approved to target and block the HER2 function in breast cancer cells. Indeed, cell colony formation inhibition was more under the combination treatment of MLN4924 and trastuzumab (Supplementary Figure 1A). Trastuzumab-induced cell cycle arrest was enhanced by MLN4924 (Supplementary Figure 1B). Accordingly, the combination of trastuzumab and MLN4924 showed lower expression of HER2 and CDK1, and higher expression of p21 (Supplementary Figure 1C). EdU staining analysis also revealed that MLN4924 combined with trastuzumab to suppress cell proliferation (Supplementary Figures 1D and E). Taken together, MLN4924 enhanced significantly the sensitivity of HER2^+^ breast cancer cells to trastuzumab.

### NAE1 overexpression in human breast cancer is associated with poor patient survival

**Figure 6:**
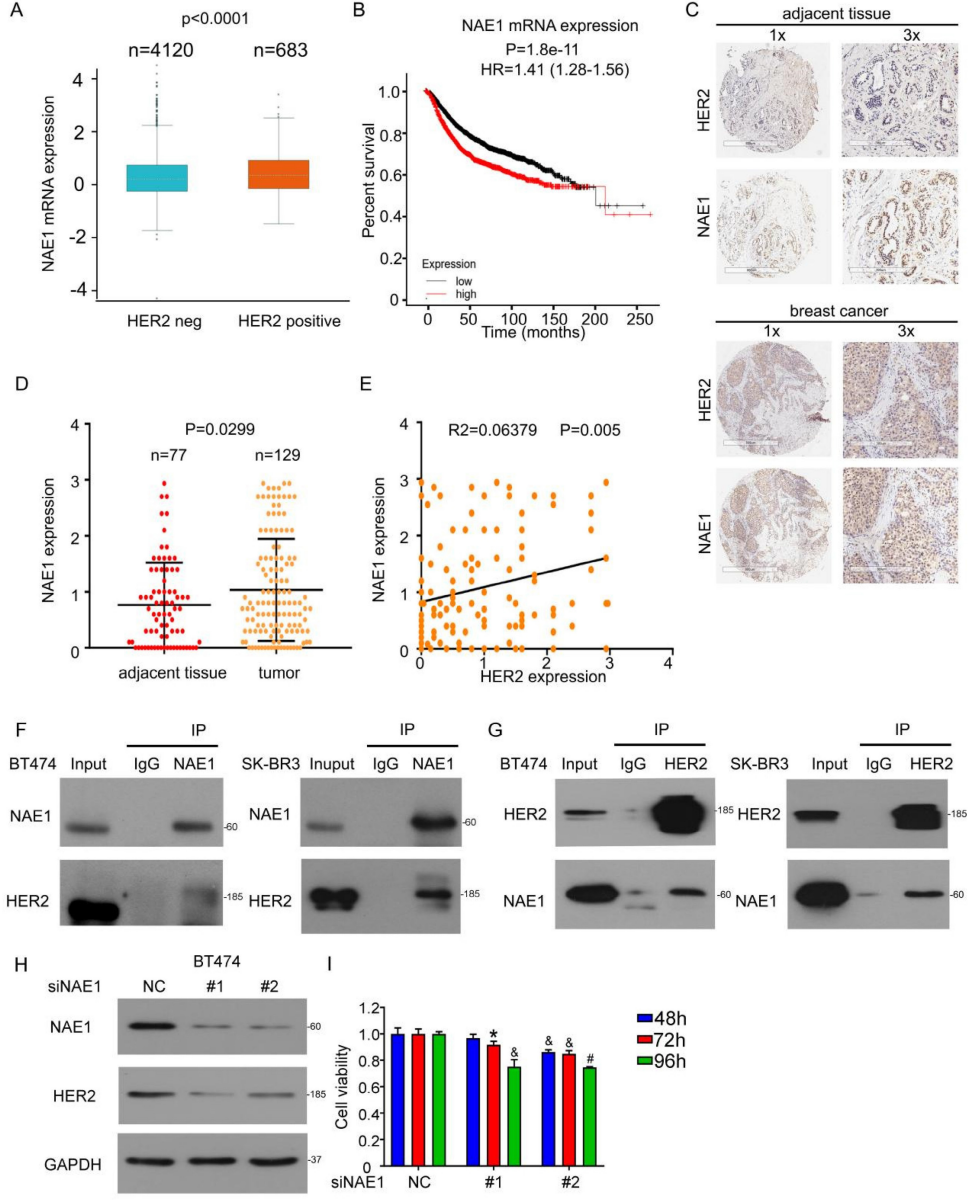
** NAE1 overexpression in human breast cancer is associated with poor patient survival. A** The mRNA level of NAE1 in human clinical specimens searching from the published microarray dataset. **B** According to NAE1 level, Kaplan-Meier curves of breast cancer patients. **C** Immunohistochemical staining using anti-HER2 and anti-NAE1 antibodies in human clinical breast cancer tissue and adjacent tissues arrays. **D** The NAE1 expressions were read and analyzed. **E** The correlation between HER2 and NAE1 expression. Cell lysis was extracted from SK-BR3 and BT474 cells. **F** Immunoblot analysis of anti-NAE1 immunoprecipitate. **G** Immunoblot analysis of anti-HER2 immunoprecipitate. **H** Cells were treated with NAE1 siRNA for 96 h, followed by western blot assay for HER2 and NAE1 expression. **I** Cell viability was evaluated by MTS assay upon NAE1 siRNA treatment.

To evaluate the potential involvement of NAE1 in breast cancer, we also analyzed the expression of NAE1 gene from published microarray data sets and found that NAE1 expression was higher in HER2^+^ human breast cancer (Figure 6A) 30 and the overexpression of NAE1 mRNA had a worse overall survival in all breast cancer (Figure 6B) 31. We further used immunohistochemical staining to analyze the expression of NAE1 in the breast cancer tissue microarray, which is composed of 129 breast cancers and 77 adjacent tissues (Figure 6C). NAE1 was highly expressed in breast cancer (Figure 6D). Interestingly, HER2 protein expression was positively correlated with that of NAE1 (Figure 6E), further suggesting the role of neddylation in the progression of clinical breast cancer. Similar to the binding of NEDD8 to HER2, Co-IP analysis also showed that NAE1 interacted with HER2 in SK-BR3 and BT474 cells (Figures 6F and G). Consequently, the knockdown of NAE1 inhibited HER2 protein expression (Figure 6H), leading to growth inhibition (Figure 6I). These findings prove that NAE1 is similar to NEDD8 in regulating HER2 expression and function.

### Neddylation-mediated breast cancer progression depends on HER2 level

**Figure 7:**
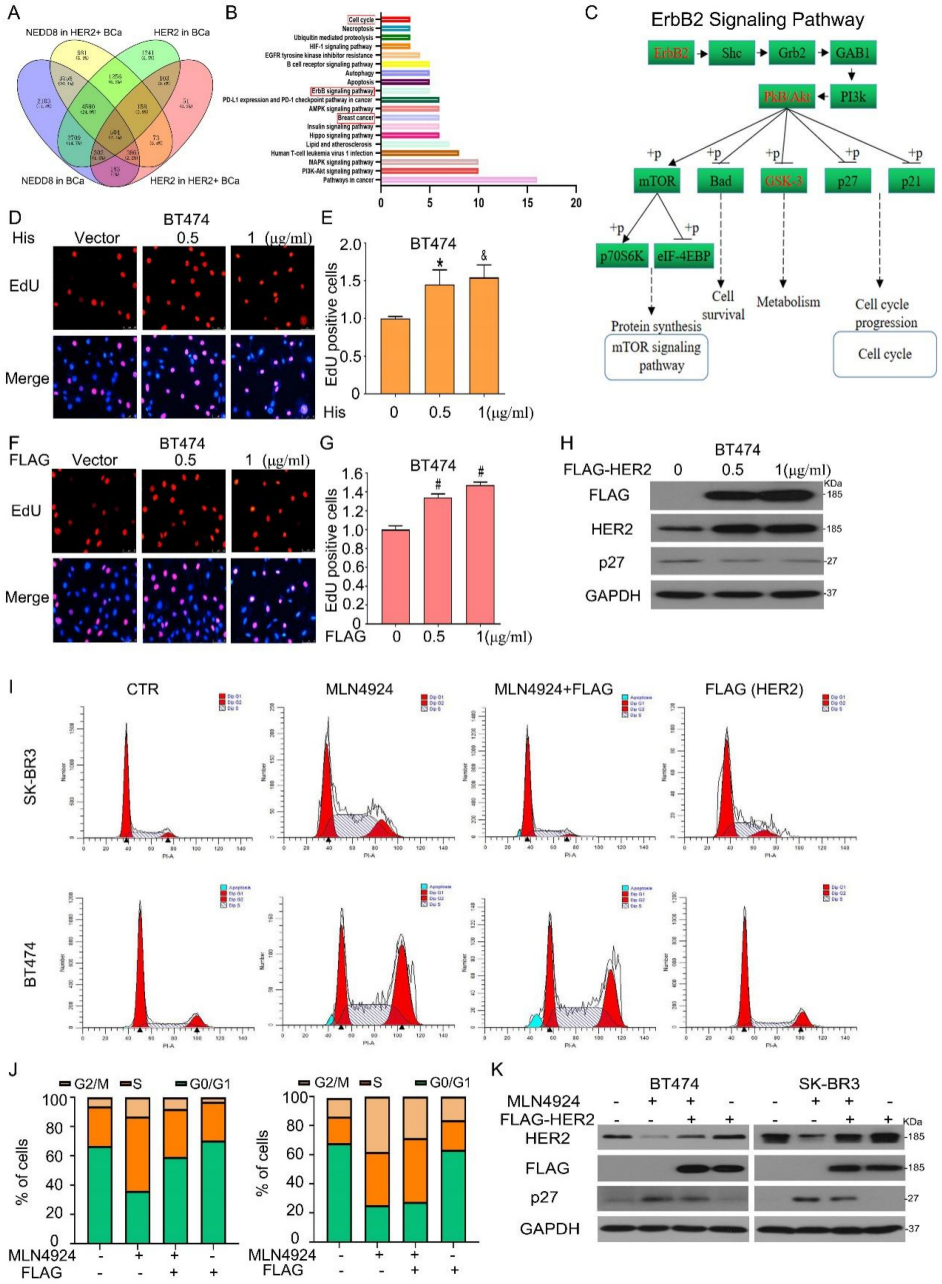
** Neddylation-mediated breast cancer progression depends on HER2 level. A** 504 genes were significantly identified which associated with NEDD8 and ErbB2 in the LinkedOmics web application (http://www.linkedomics.org). **B** The KEGG pathway enrichment of genes. **C** ErbB2 signaling pathway associated with NEDD8 was showed. BT474 cells were treated with (**D**) His-tagged NEDD8 and (**F**) FLAG-tagged HER2 for EdU staining assay. **E**, **G** The EdU stained cells was calculated. **H** Proteins were extracted using western blot analysis to test protein expression. Cells were treated with MLN4924 and FLAG-HER2, subjected to (**I,J**) flow cytometry and (**K**) western blot analysis. ^*^P<0.05, ^&^P<0.01, ^#^P<0.001.

To further examine whether HER2 is responsible for neddylation-mediated breast cancer progression, we tested the signaling pathways related with ErbB2/NEDD8 using multi-omics analysis 29. And found that 504 genes were the possible targets of NEDD8 in HER2 positive BCa (Figure 7A). Subsequently, the Kyoto Encyclopedia of Genes and Genomes pathways enrichment analysis revealed that 504 genes involved in a series of pathways. On this basis, we selected some pathways most possibility regulated by NEDD8 in HER2 positive BCa. We found that these candidate genes were enriched in ErbB2 pathway, cell cycle and breast cancer signaling pathways, which were key in BCa progression (Figure 7B). In ErbB2 pathway, we found that NEDD8 was positively correlated with ErbB2 (red) and negativity correlated with p27 (black) which cell cycle proteins (Figure 7C). Furtherly, we transduced wild-type plasmid of human NEDD8 into BT474 cells. Edu staining assay showed that NEDD8 promoted cell proliferation (Figures 7D and E). As expected, HER2 overexpression induced breast cancer cell growth faster (Figures 7F and G). This HER2-dependent process was related to decreased p27 expression (Figure 7H), which is marker of cell proliferation response. Importantly, HER2 ectopic expression blocked MLN4924-triggered G2/M phase arrest (Figures 7I and J). MLN4924-induced p27 expression was also inhibited by overexpressing HER2 (Figure 7K). Collectively, these results indicate that HER2 is required for neddylation-mediated breast cancer progression.

## Discussion

**Figure 8:**
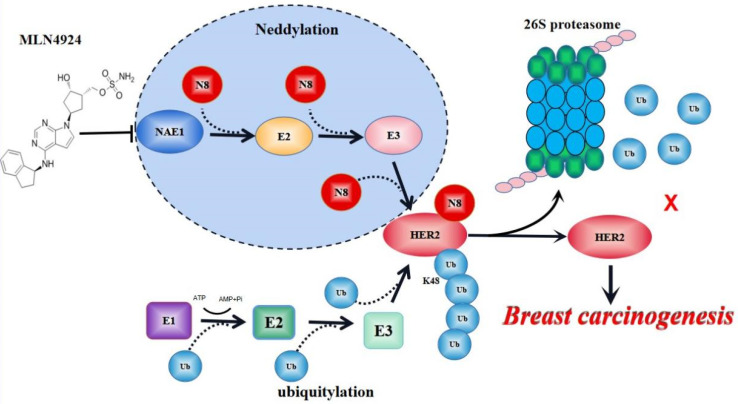
** A model for neddylation in mediating HER2 degradation and promoting breast cancer progression.** Neddylation accomplishes the modification of HER2 through N8 (N8 stands for NEDD8) attaches to HER2 protein. MLN4924 blocks this process of HER2 neddylation. While HER2 neddylation prevents it from entering the proteasome system for degradation to keep its stability, leading to breast cancer progression. Therefore, neddylated HER2 represents an unexpected function in promoting cancer growth.

PTMs are critical processes in signal transduction of glycosyl, acetyl and phosphoric acid from one protein to another. Normal cells regarded PTMs as a “switch” to judge cell proliferative and resting state, because most PTMs are reversible. In cancer cells, the inactivation of tumor suppression genes and/or oncogene activation can cause abnormal PTM status of multiple proteins for tumor growth 32,33. Crosstalk of different PTMs enhances the complexity of protein expression and functional regulation during tumor development 34. Among them, ubiquitination plays a central role in coupling to other PTMs, such as sumoylation, phosphorylation, neddylation and acetylation 35,36. By activating the UPS pathway, cells can destroy cycling proteins associated with cell cycle and signaling or damaged/misfolded proteins, or can attenuate the signal of membrane receptors, thereby maintaining a stable state 37,38. Accordingly, interference with the PTM of HER2 is an emerging anti-tumor strategy 19,20. In addition to the previous reported phosphorylation and ubiquitination, our current research suggests that neddylation is a new type of PTM for HER2 for breast cancer growth (Figure 8).

Among the ubiquitin-like protein family, NEDD8 has the highest homology with ubiquitin 21. NEDD8-mediated neddylation regulates organs development and diseases processes, including tumorigenesis. Determining the tumor-dependent substrate of neddylation is a challenge in precision medicine. Here, we provide convincing evidence that HER2 is a neddylation substrate. 1) NEDD8-cullins is bound to HER2 *in vitro*. 2) Under endogenous conditions, HER2 neddylation is detected. 3) The multimerization chain on HER2 is formed by NEDD8. 4) HER2 neddylation depends on the activating enzyme NAE1. 5) NAE1 is connected to HER2. 6) Neddylation inhibits HER2 ubiquitination. Our findings establish a new framework to understand the function and expression regulation of HER2 by neddylation in breast cancer.

We first analyze the published microarray dataset and found that NEDD8 is overexpressed in breast cancer. Our tissue microarray staining includes 77 adjacent tissues and 129 human breast cancers further strengthens the view that over-activated neddylation represents a poor prognosis of breast cancer. To further confirm the clinical role of neddylation in breast cancer progression, we evaluated the expression of a characteristic neddylation E1 (NAE1). Compared with HER2-negative breast cancer, patients with HER2^+^ tumors have higher NAE1 expression. The mRNA and protein expression of NAE1 is positively correlated with HER2 in the public dataset and our tissue microarray. These results indicate that neddylation plays an important role in the malignant progression of breast cancer, especially in the HER2^+^ subtype.

We next reveal that HER2 expression is controlled by neddylation. NEDD8 knockdown inhibits HER2 protein expression, but not change its gene transcription. Through the CHX experiment, we know that NEDD8 deletion downregulates HER2 expression by promoting its protein degradation. The use of MLN4924 to inhibit neddylation also confirmed that loss of neddylation promotes HER2 degradation. Importantly, we demonstrated that neddylation influences HER2 ubiquitination, supporting the interaction between PTMs to regulate HER2 expression. Surprisingly, excessive activation of neddylation inhibits the production of K48-linked polyubiquitin chains on HER2, while inhibition of neddylation increases HER2 ubiquitination. These findings describe in detail the kinetics of the K48 ubiquitin reaction as a typical signal of HER2 degradation. Although we found that the two core proteins in neddylation, NEDD8 and NAE1, both interact with HER2 in breast cancer cells, the details of the cooperation between NEDD8 and NAE1 in this process still need to be studied in the future. A series of HER2 truncation were generated to uncover that each domain of HER2 can be attached to NEDD8, including its ubiquitin binding site. This unexpected finding can be explained as neddylation not only regulates HER2 ubiquitination, but also regulates other forms of PTM. We used mutation technology and proved that Y1112 on HER2 is necessary for neddylation-induced ubiquitination of HER2. These findings may raise additional concerns as to whether the Y1112 mutation is a gain-of-function of HER2 in patients with breast cancer.

We finally clarify the function of neddylation in HER2 positive breast cancer cells. Inhibition of neddylation leads to tumor suppression by triggering G2/M phase arrest and apoptosis, two processes closely related to chemotherapy sensitivity. On the contrary, overexpression of HER2 reduces the activity of NAE1 inhibitors, indicating that HER2 is the main target of NAE1-mediated neddylation. These findings also support that MLN4924 is a preclinical chemical that can be used to inhibit HER2 neddylation. In addition to the single effect of MLN4924, our study also observes the synergistic effect of MLN4924 in combination with first-line drug trastuzumab in the treatment of breast cancer. These findings support further research on the effectiveness of this combination strategy in clinically relevant animal models.

In summary, we identify neddylation as a novel modification of HER2 and reveal that HER2 neddylation is positively correlated with the development and poor prognosis of breast cancer. Our findings may open up a new path for precision therapy by targeting the neddylated HER2 protein in patients with breast cancer.

## Materials and Methods

### Cell culture

HER2-positive breast cancer cells BT474 and SK-BR3, and HEK293T cells were purchased from American type cultures (Manassas, VA, USA). The above cells were cultured in 37 °C and 5% carbon dioxide incubator. RPMI-1640 medium contained 10% FBS (Fetal bovine serum) was for BT474. SK-BR3 were cultured on McCoy '5A medium with 10% FBS (Australian fetal bovine serum), and DMEM medium containing 10% FBS was for HEK293T cells.

### Antibodies and chemicals

MLN4924 (#S7109), MG132 (#S2619), Cycloheximide (CHX) (#S7418), Trastuzumab (anti-HER2) (A2007) were provided from Selleck Chemical (Houston, Houston, Texas, USA), antibody: Anti-HER2 (#2165), anti-NEDD8 (#19E3), anti-K48-Ub (#12805), anti-FlAG (#14793), anti-His (#12698), anti-PARP (#9532), Anti-Bcl-2 (#15071), anti-p27 (#5174), anti-p21 (#2947), anti-GAPDH (#5174) were purchased from Cell Signaling Technologies, Inc. (Beverly, MA). Anti-CDK1 (AB201008), and anti-NAE1 (APPBP1) (AB187142) were purchased from Abcam (Cambridge, Ma).

### Clinical selection

We selected 129 human breast cancer tissue arrays and adjacent tissue arrays from 77 patients, which were from Shanghai Outdo Biotechnology Company (Shanghai, China). Tissue arrays abide by the Declaration of Helsinki principles and were approved by the ethics committee of Shanghai Outdo Biotech Company (Shanghai, China). Anti-HER2 (#2165) and anti-NEDD8 (#19E3) were used for immunolabeling of paraffin-embedded sections to evaluate the expression of HER2 and NEDD8 in cancer and adjacent tissues.

### Transfection of plasmids and siRNAs

The plasmids and RNA interfering assays were performed according to our previous reports 39,40. We constructed and purchased plasmids from GeneChem (Shanghai, China). The human HER2 plasmids (CMV-MCS-3FLAG-SV40-Neomycin) and NEDD8 plasmids (CMV-MCS-SV40-Neomycin), HER2 mutant plasmids (NM_004448(Y1112F)), and HER2 truncated plasmids (1-653aa), HER2 (654-1255aa), HER2 (720-987aa), HER2 (988-1255aa) and their control plasmids were for our plasmid transfection experimen. SK-BR3 and BT474 were digested and placed in 6-well plates or dishes. After cell adherence, the cells were transfected with plasmid concentration of 0.5-1 μg/mL. Taking 2ml medium system as an example: 500 μL opti-MEM +2 μl P3000 +2 μl lipofectamine 3000+2 μg plasmid. After 48 or 72 hours of transfection, the transfection efficiency was determined to complete the next experiment. The expression level of target gene was down-regulated by siRNA interference experiment. In short, SK-BR3 and BT474 were inoculated in culture dishes for 24 h, then the mixture of RPMI opti-MEM, lipofectamine RNAiMAX and siRNA were cultivating cells for 72 h. The NEDD8 siRNAs and NAE1 siRNAs were purchased from RiboBio (Guangzhou, China). The siRNA sequences are listed below:

- NEDD8 siRNA-1: 5'-AGAAGACAGCAGCTGATTA-3';
- NEDD8 siRNA-2: 5'-AGAGGAGGAGGTGGTCTTA-3';
- NAE1 siRNA-1: 5'-GAGGCACAATTCCTGATAT-3';
- NAE1 siRNA-2: 5'-GTTACGGGCTGTTGATAGA-3'.

### RNA extraction and Real-time RT-PCR assay

According to our previous reports 41, The RNAiso plus (TakaRa Biotechnology, Dalian, China) was used to extract total cellular RNA of SK-BR3 and BT474, then the RNA purity and concentration were determined at 260:280 nm. After diluting rnase-free water to the same concentration,we used priMEScript ™RT master mix (Takara Biotechnology, Dalian, China) to reverse 1 μg of total RNA and synthesized the first strand cDNA as instructed. The Real-time quantitative PCR was used to detect HER2 and GAPDH mRNA levels. Three separate experiments were conducted.

PCR primes are as following:

- ERBB2: F: 5' AACTGCACCCACTCCTGTGT 3';
- ERBB2: R: 5'TGATGAGGATCCCAAAGACC 3';
- GAPDH: F: 5'TCCCATCACCATCTTCCA3';
- GAPDH: R: 5' CATCACGCCACAGTTTCC3'.

### Cell proliferation assay

According to our previous reports 42-44, cell viability, colony formation, and EdU staining were used to indicate cell proliferation. MTS Kit (#G3581, Promega, Madison, WI, USA) was apply for testing cells' dynamic, in brief, SK-BR3 and BT474 were planted in 96-well plates overnight, then the cells were treated with drug or siRNA for 48, 72, 96h, followed by MTS assay. For colony formation, monitoring the long-term proliferation ability of cells. Firstly, SK-BR3 and BT474 were placed in 6cm dishes and treated with MLN4924 or Trastuzumab for 2 days, then the cells were re-plated in 6-well plates and showed in crystal violet. EdU staining was like we reported before. The cells were digested and seeded at chamber slide overnight, incubating drugs or siRNA for 48 h or 72 h, Cell-Light™ EdU Apollo 567 *In vitro* Kit (Cat number: C10310-1, RiboBio, Guangzhou, China) were performed to detect the cell proliferation. All experiments were repeated three times.

### Cell cycle and apoptosis

SK-BR3 and BT474 were treated with MLN4924 for 48 h or NEDD8/control siRNA for 72 h to be prepared for the cell cycle and apoptosis. For cell cycle assay, the cells were collected to fix in 4 °C 70% anhydrous ethanol and PBS washed it once, then added the compound of 50 μg/ml PI, 100 μg/ml RNaseA and 0.2% Triton-X-100 to the cell suspension, detected by flow cytometry. Apoptotic treatment of the specimen followed the same cell cycle, the difference is each group was re-suspended with Annexinv-FITC binding buffer 500 μL, Annexinv-FITC 5 μL and PI mixture 5 μL, respectively, incubating for 30 min, the stained cells were analyzed by flow cytometry.

### Western blot and co-IP assay

The detection method is the same as the one we reported earlier 45. Cell lysis solution containing the treated cells were sapareting by SDS-PAGE and then transfered to PVDF membranes. After 1 h of 5% non-fat milk incubating and the spacial primary antibodies incubated for the night, then the second antibody was binding for 1 h, lastly, the corresponding protein-antibody complex was analyzed with an ECL way and then analyzed with X-ray film. The Co-IP experiment was firstly making the magnetic beads and antibody mixture incubating for 16-24h, the following was binding the protein to the antibody about 1-2 h at 4 °C. Finally, washing the mixture three times with PBST, detecting it by immunoimprinting.

### Immunofluorescence assay

Based on previous research 46, SK-BR3 was transfected with flag-HER2 or His-NEDD8 plasmids and exposed for MLN4924 about 48 h, then fixed with 4% paraformaldehyde for 30 min, infiltrated 0.5% triton-x for 10 min, and 5% BSA sealed the cells for 30 min, then the primary antibody was added to the cells at 4 °C overnight. The following was the second antibody with the cells for 1 h in the dark after rinsing with PBS for three times.We used DAPI (Abcam, # AB104139) to dye nuclerr, and cells were observed by the confocal microscope (LeicaTCSSP8).

### Immunohistochemical staining and intensity analysis

Tumor sections were immunostained with MaxVision kit (MaixinBiol) according to the standard technique we reported previously 44. Then the tissue samples were immunohistochemically treated with HER2 and NEDD8. Each slide was added with 50μlMaxVisionTM reagent and dyed with 0.05% diaminobenzidine and 0.03% hydrogen peroxide in 50 mM tris-hcl (pH7.6). The slides were multicolor with hematoxylin and the primary antibody was using DAB to determine.

### *In vitro* neddylation assay

The purified proteins expressed HER2 were purchased (Cat. No. HE2-H5225, Acrobiosystems, Delaware, USA). Neddylation of HER2 (1 μg in each sample, 37 °C, 1 h) was detected with a commercial kit (Cat. No. BML-UW0590, Enzo Life Sciences, Farmingdale, NY, USA) *in vitro*. The neddylation of HER2 was tested by IB according to the manufacturer's instructions.

### Statistical methods

We apply t test or ANOVA to evaluate the statistical significance of the data, and the kaplan-Meier method and ANOVA were used to analyze the survival curve. Simultaneously, pearson correlation analysis was applied for determining the correlation between HER2 and NEDD8. P value is less than 0.05 was identified as statistically significant. Our statistical significance as show below: *P < 0.05, &P < 0.01, #P < 0.001, and the statistical data were expressed as the mean ± standard deviation of three independent experiments.

## Supplementary Material

Supplementary figures.
