# Supplementary material for: Neddylation of HER2 Inhibits its Protein Degradation and promotes Breast Cancer Progression
Source: Int J Biol Sci. 2023 Jan 1;19(2):377–92. doi: 10.7150/ijbs.75852 (PMC9830515; doi:10.7150/ijbs.75852)
Supplement: Supplementary file 1 — Supplementary figures. [file ijbsv19p0377s1.pdf]

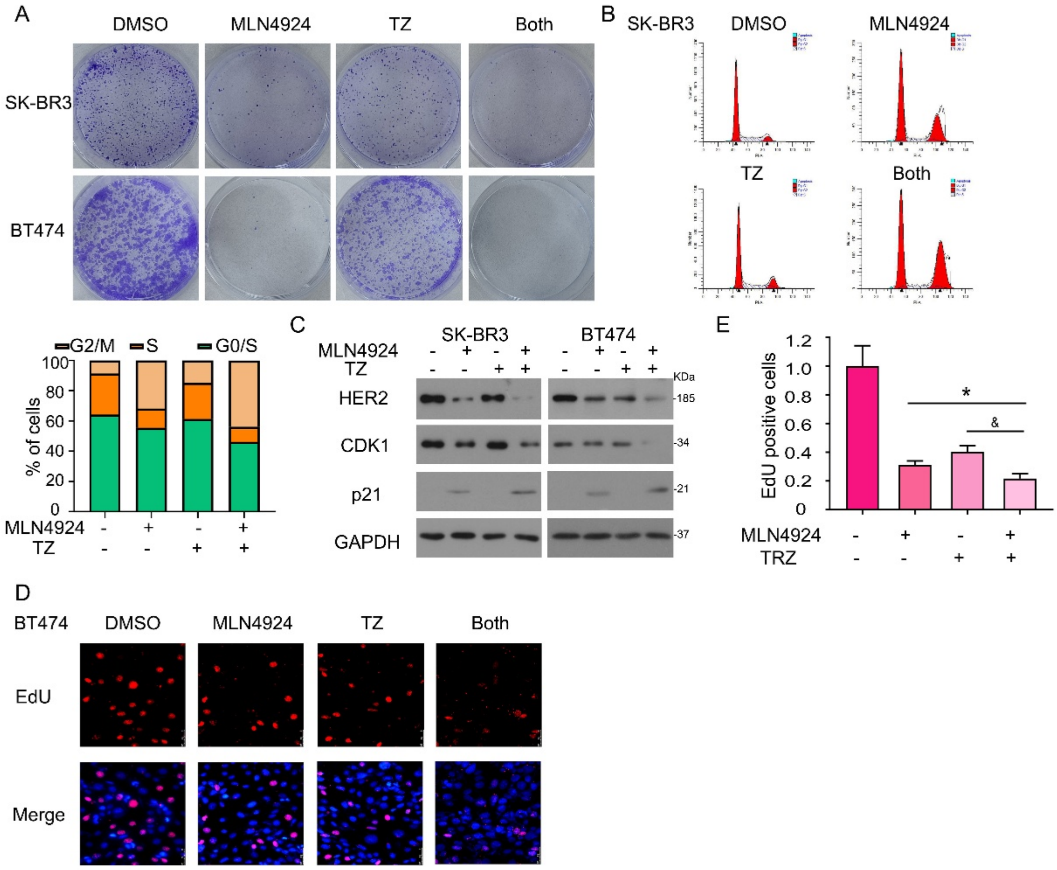

Supplementary Figure 1. The combination of MLN4924 and trastuzumab suppresses HER2 expression and cell growth. **A** SK-BR3 and BT474 cells were treated with MLN4924 (0.5 μM) and/or trastuzumab (TZ)(50 μg/ml) for colony formation. SK-BR3 were treated with MLN4924 and/or trastuzumab (TZ), followed by analyzing (**B**) cell cycle progression and (**C**) related proteins expression, including HER2, CDK1 and p21. Additionally, the combined treated cells were subjected to (**D**) EdU staining and (**E**) the positived cells were calculated. $^{*}P<0.05$, $^{\&}P<0.01$.

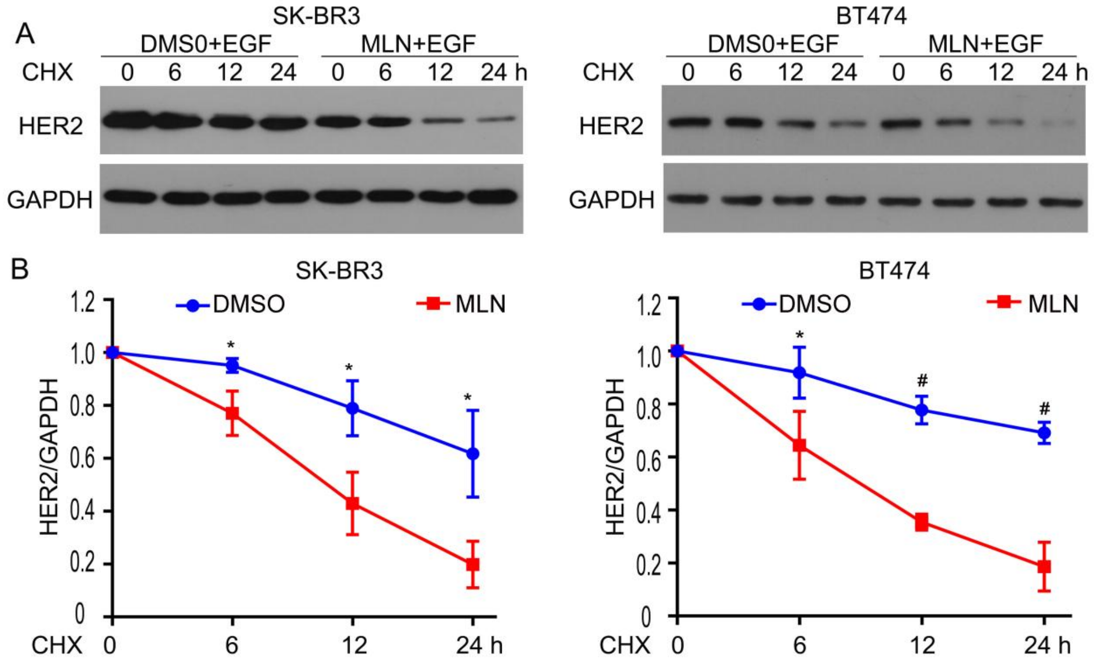

Supplementary Figure 2. MLN4924 accelerates HER2 protein degradation. A. SK-BR3 and BT474 cells were treated with DMSO/MLN4924, EGF and CHX (50 μg/ml) for HER2 protein expression. **B.** The bands of HER2/GAPDH quantified by densitometry with Image J. $^{*}P<0.05$, $^{\#}P<0.001$.

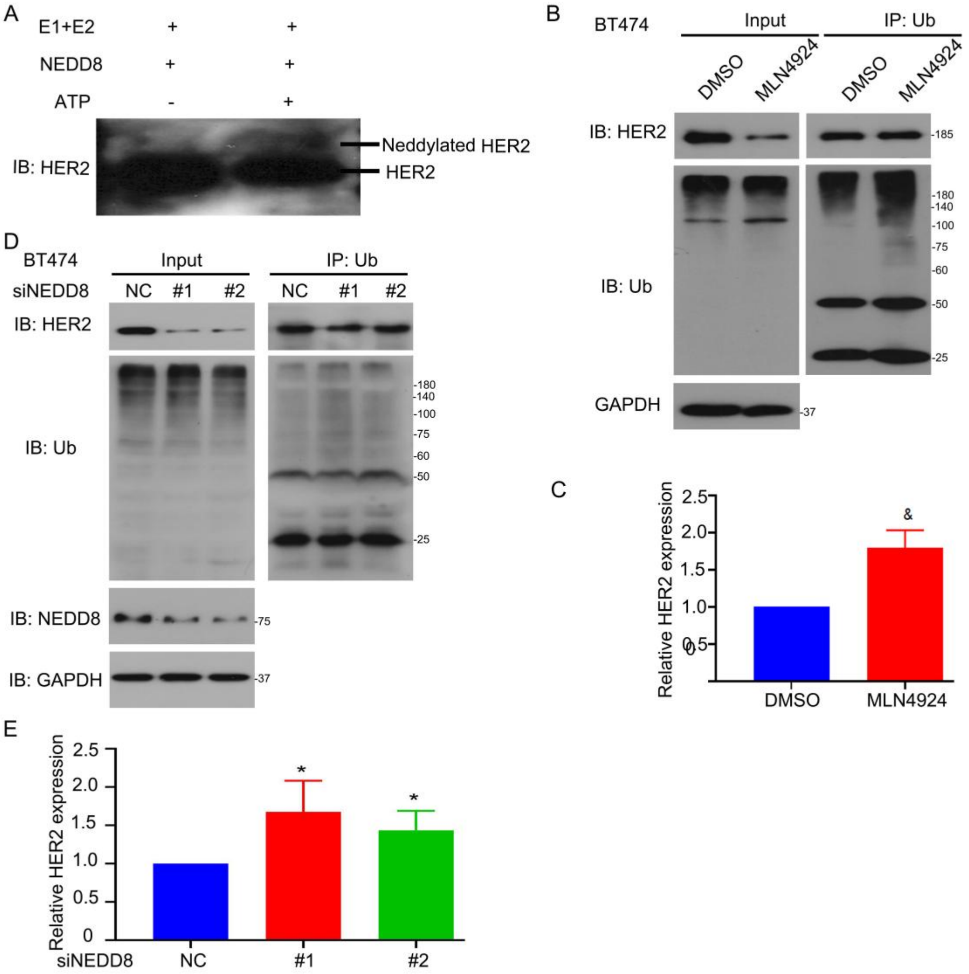

Supplementary Figure 3. HER2 is a neddylation substrate and neddylation inhibits HER2 polyubiquitination. A. The purified proteins at 37° C in the absence or presence of ATP for 1 h. The samples were then subjected to IB with an antibody against HER2. BT474 cells were treated with (**B**) MLN4924 and (**D**) NEDD8 siRNA, respectively. Immunoblot analysis of anti-ubiquitin immunoprecipitate for HER2 expression. **(C, E)** The relative expression of HER2 quantified by the treated group (HER2 (IP) : Ub (IP) : HER2 (Input)) : control group (HER2 (IP) : Ub (IP) : HER2 (Input)). $^{*}P<0.05$, $^{\&}P<0.01$.
